# Contribution of sex on the underlying mechanism of the gambling disorder severity

**DOI:** 10.1038/s41598-020-73806-6

**Published:** 2020-10-30

**Authors:** Susana Jiménez-Murcia, Roser Granero, Mónica Giménez, Amparo del Pino-Gutiérrez, Gemma Mestre-Bach, Teresa Mena-Moreno, Laura Moragas, Marta Baño, Jéssica Sánchez-González, Marta de Gracia, Isabel Baenas-Soto, S. Fabrizio Contaldo, Eduardo Valenciano-Mendoza, Bernat Mora-Maltas, Hibai López-González, José M. Menchón, Fernando Fernández-Aranda

**Affiliations:** 1 Ciber Fisiopatología Obesidad y Nutrición (CIBERobn), Instituto Salud Carlos III, Madrid, Spain; 2 Department of Psychiatry, Hospital Universitari de Bellvitge, L’Hospitalet de Llobregat, C/Feixa Llarga S/N, C.P. 08907 Barcelona, Spain; 3 Department of Clinical Sciences, School of Medicine, Universitat de Barcelona - UB, L’Hospitalet de Llobregat, Barcelona, Spain; 4 Psychiatry and Mental Health Group, Neuroscience Program, Institut D’Investigació Biomèdica de Bellvitge - IDIBELL, L’Hospitalet de Llobregat, Barcelona, Spain; 5 Department of Psychobiology and Methodology, Autonomous University of Barcelona, Barcelona, Spain; 6 Ciber Salud Mental (CIBERsam), Instituto de Salud Carlos III, Madrid, Spain; 7 Department of Public Health, Mental Health and Perinatal Nursing, School of Nursing, University of Barcelona, Barcelona, Spain

**Keywords:** Translational research, Psychology

## Abstract

Significant increasing prevalences have been observed in gambling disorder (GD) in the last decades. This study analyzed the underlying mechanisms of the gambling severity with path analysis (implemented through Structural Equation Modeling, SEM), and assessed the potential moderator effect of the patients’ sex. A sample of n = 512 treatment-seeking patients was assessed for sociodemographics and clinical state previously to the treatment. Results obtained in two separate SEM (for men and women) revealed differences in the direct effects and the mediational links. Among the male subsample, higher GD severity was directly related to the higher cognitive bias and the younger age of onset of the problematic gambling, while impulsivity levels and age of onset achieved an indirect effect on the disordered gambling mediated by the cognitive bias. Among females, GD severity was directly increased by younger age of onset, higher cognitive bias and lower self-directedness, while lower socioeconomic positions, and higher levels in harm avoidance achieved an indirect effect on the gambling severity mediated also by the distortions related to the gambling activity. These results provide new empirical evidence for a better understanding of the GD etiology, suggesting that the underlying complex links mediating the GD severity are strongly related to the patients’ sex. The results can also contribute to design more effectiveness and precise therapy programs of patient-centered care.

## Introduction

Gambling is a very common social activity, initially practiced as a leisure action, than in non-desirable percentages may suppose critical situations for gamblers. In Europe, i.e., problem gambling rates up to 3%, while in some non-European countries this percentage increases up to the 6%^1,2^. Gambling disorder (GD) is considered an activity with harmful public health consequences, which are mainly underestimated^3^.

The latest classification change of GD as a “substance-related and addictive disorder” in the last edition of the *Diagnostic and Statistical Manual of Mental Disorder* (DSM-5)^4^ has important implications for understanding the nature of the disorder and the associated factors that may maintain and/or make worse the behavior of gamblers. In this sense, problem gambling involves not only social, economic, family, or occupational costs but also a significant personal burden and impact on clinical, affective-cognitive, and personality spheres of patients. Multiple gambling subtypes exist, being the differentiation into non-strategic versus strategic (based on the level of the influence of change) the one used during recent years in the clinical and research areas^5^. Chance-based games are categorized within the non-strategic category, which includes games which involve little deliberation or skill and the potential result is 100% dependent on chance (such as slot-machines, bingo or lotteries). On the contrary, skill-based games are grouped within the strategic category, which includes games that allow gamblers to attempt to use game-related knowledge to predict the potential results (such as dice, poker and other cards, betting on sports events or races, craps, or the stock market).

Even though there have been multiple etiological and risk factors-related studies to estimate the nature and scope of GD^6–8^, efforts should not just be directed at the etiology of the disease, but also to explain the factors affecting the maintenance and/or the severity of the disorder, in order to better establish treatment protocols to change the course of the disease.

Among sociodemographic variables, age has been related to psychopathological and other clinical aspects associated with pathological gambling^9^. Age has specifically been related to cognitive distortions in GD, in the sense that younger patients have a lower illusion of control^10^. Gambling-related cognitive distortions are influenced by subjective reward value, interoception, and risk prediction^11^, and appear as a hallmark for GD^12,13^ that has been associated with GD severity^14^. A very recent study postulated that cognitive distortions were directly related to baseline psychiatric symptomatology in gambling^15^. There are other neuropsychological variables involved in GD that have been evaluated in order to define prevention approaches. Personality-related traits, such as neuroticism, impulse, self-directness, or harm-avoidance, among others, appear clearly dysfunctional in GD^8,16–22^, and even some of these factors seem to facilitate vulnerability to GD^23^.

Affective aspects are also involved in GD and its severity. It is well known that GD and depressive symptoms can co-occur^24–26^. A direct effect of depressive symptomatology on gambling-related problems has also been reported^27^. Some studies point out that negative and/or depressive mood states appear as moderators in the relationship between dysfunctional cognitions and severity of the disorder^28^, while other authors found gambling-related cognitive distortions mediating the relationship between depression and severity^29^. Also, it has been reported that adults with the impulsiveness as a personality trait and depression showed more gambling problems than adults without^8^.

There is a recent study about trajectory of gambling severity in the absence of treatment seeking^30^, pointing out a progression of self-directedness change in the sense that participants with more severe gambling problems at baseline reported greater reductions in their gambling severity over time. The authors explained this apparent contradictory result by the fact that all gamblers were motivated to quit or diminish their gambling activities, and that in most severe cases they could be experiencing greater recognition of gambling consequences. This may be translated into a more immediate urgency to change the pathological behavior, with significant self-directed changes in gambling severity.

All in all, the role of all these factors underlying gambling severity has not been comprehensively studied in the same model. In this sense, with the growth of females participation in gambling activities^31^, sex appears as an increasingly important factor within the evaluation of aspects underlying severity of GD. In treatment-seeking individuals with GD, sex differences have been already reported^9,22,32–34^. However, very few studies evaluate the profile factors for gambling in females^18,35^.

Bearing in mind the sex differences in GD profiles, gambling severity for females and males looking for treatment does not necessarily be mediated by the same factors. The association of personality factors (impulsivity trait) with age and sex in GD found in previous studies highlights the need to detect the aspects that are conditioning severity and the mediating effects that are present^36^. In terms of GD severity characterization specifically, there are no studies assessing the mediators of gambling severity in males and females separately.

In non-treatment seeking populations, the effects of different factors on GD severity have hardly been explored by means of a path analysis. A recent study has explored the mediating effect of alexithymia on gambling severity^37^. Another path analysis study revealed gambling severity increase via depressive symptoms^38^; but none of these studies were stratified by sex. In treatment-seeking subjects, there are very few studies using pathways approaches. Among them, a previous study evaluated the relationship between reward and punishment sensitivity and GD^39^, showing that both variables were positively and directly associated with increased gambling severity. Another study evaluated patient sex and personality traits in the pathways, explaining the age of onset of GD and its clinical profile (severity of gambling behavior and depressive symptoms)^40^, and found that sex had a direct effect on GD onset and depression symptoms, in the sense that males initiated GD earlier and reported fewer depression symptoms. However, these studies did not evaluate the direct or indirect mediating effects of different factors in severity, by sex.

The evaluation of the role of the variables involved in the pathways explaining GD severity grades taking sex into account seems essential to determine prospective treatments taking into account the idiosyncrasy of each group.

### Objectives

The aim of the study was to assess, through pathways analysis, the underlying mechanisms of GD severity, considering the direct and indirect (mediational) effects between a broad set of variables including sociodemographic, personality, and other clinical measurements, and to value the potential role of patient sex as a moderator variable. To our knowledge, this is the first study examining differences in the pathways that explain the gambling severity between males and females in a clinical based sample.

## Material and methods

### Participants and procedure

The sample of this work was recruited for a multicenter research project aimed at examining risk factors for GD severity among an adult population of individuals with gambling impairment. The participants were treatment-seeking patients from different clinical settings specialized in the treatment of gambling problems and other behavioral addictions. The sample comprised *n* = 512 patients recruited between July 2016 and October 2016. Inclusion criteria in the study were aged > 18 years, reporting gambling related problems, and adequate education and cognitive capacity to complete the self-report measures of the study. Only patients who sought treatment for GD as their primary health concern were admitted to this study. All the treatment-seeking patients for gambling related problems during the recruitment data were included in the study, and it was not required the presence of a full diagnosis of GD based on a diagnostic taxonomy (both problematic gamblers and GD were analyzed).

The sample of the study was composed mostly by males (*n* = 473, 92.4%), with married (*n* = 245, 47.9%) or single (*n* = 198, 38.7%) marital status, low education levels (mostly, primary; *n* = 293, 57.2%), low social indexes (*n* = 289, 56.4%), and gambling activities onset at a mean age 21.0 years (SD = 8.9). The mean chronological age was 43.0 years (SD = 13.5).

As regards lifespan gambling activities, the higher prevalences were for slot-machines (*n* = 380, 74.2%), lotteries (*n* = 313, 61.1%), football betting pools (a form of gambling where gamblers pay a fixed price into a pool and then make a selection on the outcomes related to the football league, and winner’s payoff depends on the number of gamblers and the number of winners) (*n* = 247, 48.2%), and bingo (*n* = 188, 36.7%). Considering the classification of the gambling preferences, the most prevalent was reporting both non-strategic and strategic games (*n* = 322, 62.9%), followed by only non-strategic (*n* = 138, 27.0%) (only strategic gambling was reported by *n* = 52, 10.2%).

### Measures

*Diagnostic Questionnaire for Pathological Gambling according to Diagnostic and Statistical Manual of Mental Disorders (DSM) criteria*^41^. This is a self-report used to assess the presence of the GD through 19 items developed for measuring the diagnostic criteria defined in the DSM-5 taxonomy^4^. The Spanish adaptation of the scale was used (which achieved good psychometric properties: α = 0.81 for general population and α = 0.77 for GD clinical sample)^42^. This work analyzed the total number of DSM-5 criteria for GD as a measurement of the gambling severity (internal consistency in the sample was very good, α = 0.84).

*Gambling-Related Cognitions Scale* (GRCS)^43^. This self-report contains 23 items to assess the level of cognitive bias in five primary cognitive dimensions (gambling related expectancies, illusion of control, predictive control, perceived inability to stop gambling, and interpretative bias). This study analyzed the GRCS total score, obtained by summing the score for each item, as a measurement of the global cognitive bias related with the gambling severity (the internal consistence in the sample was excellent α = 0.95).

*Temperament and Character Inventory-Revised (TCI-R)*^44^. This questionnaire contains 240 items for measuring personality traits structured into 7 personality dimensions: 4 are dimensions related to temperament (novelty seeking, harm avoidance, reward dependence, and persistence), and 3 were character dimensions (self-directedness, cooperation, and self-transcendence). For the current study, the Spanish version of TCI-R was used^45^. Harm avoidance (α = 0.73 in our sample) and self-directedness (α = 0.82 in our sample) were the variables studied here.

*Impulsive Behavior Scale (UPPS-P)*^46^. This questionnaire includes 59 items developed for assessing five impulsivity factors: lack of perseverance, lack of premeditation, sensation seeking, negative urgency, and positive urgency. This work uses the Spanish adaptation of the tool, which has obtained adequate psychometrical properties^47^. The internal consistency in the study was between α = 0.84 for lack of perseverance and α = 0.95 for positive urgency.

*Patient Health Questionnaire (PHQ-9)*^48^*.* This is a brief questionnaire based on 9 items for screening depression-related symptoms level, with good psychometrical properties in different settings^49^. The total score (with a consistency of α = 0.92 in this work) was evaluated in this study.

*Stressful Life Events (SLE)*. An ad-hoc questionnaire, created for the current research project, was used to evaluate the incidence of SLE lifespan. This questionnaire considers 31 life events that could disturb individuals and cause substantial change and readjustment (including: violent behaviors, moving to a new city or to a new country, loss of relative ones, severe illness, conflicts with law, severe financial problems, sexual problems, getting married, divorces, victim of accidents, severe problems with family or friends, unwanted pregnancy, abortion, birth of a child, problems in parenting, job changes/promotions/losses, extramarital sex,). For each SLE, respondents are asked to report whether it occurred (yes–no), their age when the event occurred and the degree of its influence (zero, moderate, some, or considerable). The total number of lifetime SLE was analyzed in this work (internal consistency in the sample was α = 0.78).

*Other clinical and sociodemographic variables.* The other measures of the study were registered with a semi-structured clinical interview (self-report format). It covered different sociodemographic characteristics, some of them gambling related variables (i.e. gambling preferences and age of onset of the gambling activities). The social position index was obtained using Hollingshead’s algorithm^50^.

All the measures analyzed in the study correspond to the assessment at the arrival of patients at the treatment setting, before starting the therapy. The information of the semi-structured clinical interview was collected by psychologists and psychiatrists with extensive experience in behavioral addictions, who also helped the patients to complete the self-report questionnaires (guaranteeing that the items were all answered and that no problems had occurred due to lack of understanding).

### Ethics

This work was performed in accordance with the Helsinki Declaration of 1975 as revised in 1983, and it was approved by the Ethics Committee of University Hospital of Bellvitge (reference number PR095/16). Written informed consent was obtained from all the participants in the study. All the data analyzed in this work correspond to the first assessment before the patients began the therapy.

### Statistical analysis

Statistical analysis was carried out using Stata16 for windows. Firstly, the differences between men and women in sociodemographics and the clinical variables of the study was based on chi-square test (χ^2^) for comparing the proportions registered in the categorical measures and on T-TEST procedures for comparing the means obtained in the quantitative features.

Next, the bivariate correlation between the variables of the study was estimated using Pearson’s correlation. Due the strong association between the statistical significance of these coefficients and sample size (low coefficients tend to achieve significance in large samples and high coefficients do not achieve significance in small samples), the effect size was interpreted based on the |*R*|-estimation: low-poor |*R*|> 0.10, mild-medium for |*R*|> 0.24, and high-large for |*R*|> 0.37 (these thresholds correspond to a Cohen’s-*d* of 0.20, 0.50 and 0.80, respectively)^51^.

Finally, path-analysis, implemented using SEM, estimated the magnitude and significance of the associations within the set of variables considered in this work, including mediational links (direct and indirect effects)^52^. The maximum-likelihood (MLE) method of parameter estimation has been used, and goodness-of-fit was evaluated using the standard statistical indexes: the root mean square error of approximation (RMSEA), the Bentler Comparative Fit Index (CFI), the Tucker-Lewis Index (TLI), and the standardized root mean square residual (SRMR). Adequate model fit was considered when the following criteria were met^53^: RMSEA < 0.08, TLI > 0.90, CFI > 0.90, and SRMR < 0.10. The global predictive capacity of the model was measured by the coefficient of determination (CD). In this study, due the large number of variables to be considered in the path analysis, a latent variable measuring the impulsivity construct through the UPSS-P scores was defined.

## Results

### Sample description

Table 1 shows the comparison by sex for the variables analyzed in the study. The subsample of males included a higher proportion of participants in a single marital status and belonging to lower social position indexes. Compared to females, males were also younger and reported early onset of the gambling behaviors, as well as higher number of DSM-5 criteria for GD, higher impulsivity levels (in lack of perseverance, sensation seeking, and positive urgency), a lower level in the self-directedness personality trait, and a lower SLE lifespan.

**Table 1:** Descriptive for the sample (n = 512).

	Women		Men		*p*
	(*n* = 39)		(*n* = 473)		
	*n*	*%*	*n*	*%*	
**Marital status**					
Single	10	25.6%	188	39.7%	.013*
Married	18	46.2%	227	48.0%	
Divorced	11	28.2%	58	12.3%	
**Education**					
Primary	22	56.4%	271	57.3%	.552
Secondary	8	20.5%	122	25.8%	
University	9	23.1%	80	16.9%	
**Employment**					
Unemployed	18	46.2%	190	40.2%	.465
Employed	21	53.8%	283	59.8%	
**Social position index**					
High	0	0.0%	8	1.7%	.022*
Mean-high	5	12.8%	26	5.5%	
Mean	6	15.4%	70	14.8%	
Mean-low	14	35.9%	94	19.9%	
Low	14	35.9%	275	58.1%	
	Mean	SD	Mean	SD	p
Chronological age (years)	48.31	11.98	42.54	13.49	.010*
Age of onset (years)	25.99	11.40	20.62	8.52	.001*
DSM-5 total criteria	6.05	2.80	7.22	1.94	.001*
GRCS: total cognitive bias	62.77	31.38	65.53	32.81	.613
PHQ: depression total	8.23	7.34	7.47	6.78	.501
UPPS-P: lack of premeditation	22.13	6.55	23.72	7.09	.177
UPPS-P: lack of perseverance	16.67	5.14	21.37	6.13	.001*
UPPS-P: sensation seeking	21.72	7.47	26.73	8.28	.001*
UPPS-P: positive urgency	27.28	10.37	31.65	11.56	.023*
UPPS-P: negative urgency	30.31	8.65	32.32	8.17	.143
TCI-R: harm avoidance	102.87	13.78	98.91	12.57	.061
TCI-R: self-directedness	142.77	26.21	131.84	23.56	.006*
SLE: lifespan total	8.18	4.67	5.74	4.36	.001*

DSM, Diagnostic and Statistical Manual of Mental Disorders; GRCS, gambling-related cognitions scale; PHQ, patient health questionnaire; SLE, stressful life events; TCI-R, temperament and character inventory-revised; UPPS-P, impulsive behavior scale; SD, standard deviation.

*Bold: significant comparison (0.05 level).

Regarding the gambling activities, differences between sexes were found for football betting pools [this activity was highly reported by men compared to women (50.5% *versus* 20.5%); χ^2^ = 13.00, *p* = 0.001) and the overall gambling preference [a higher prevalence of strategic mode was found among male sex compared to female (10.6% *versus* 5.1%); χ^2^ = 6.29, *p* = 0.043).

### Correlation analysis

Table 2 contains the bivariate correlation matrix for the variables of the study. The estimations obtained among the male subsample (upper part of the Table) showed that the GD severity (measured as the number of DSM-5 criteria) was higher for participants with higher depression level, higher cognitive bias, higher positive and negative urgency impulsivity, lower self-directedness, and younger age of onset. Higher depression symptom level and higher impairing cognitive bias correlated with higher impulsivity levels and more dysfunctional personality traits.

**Table 2:** Correlation matrix between the variables of the study (n = 512).

		1	2	3	4	5	6	7	8	9	10	11	12	13	14
1	DSM-5 total criteria for GD	–	**.24**^**†**^	**.32**^**†**^	.23	.22	.18	**.28**^**†**^	**.33**^**†**^	.13	− **.26**^**†**^	− .17	− **.32**^**†**^	.07	.03
2	PHQ: depression total	***.36***^***†***^	–	**.45**^**†**^	**.30**^**†**^	**.39**^**†**^	.19	**.40**^**†**^	**.43**^**†**^	**.29**^**†**^	− **.51**^**†**^	− .04	− .10	.08	− .04
3	GRCS: cognitive bias total	***.33***^***†***^	*.16*	–	**.31**^**†**^	**.31**^**†**^	**.24**^**†**^	**.33**^**†**^	**.31**^**†**^	.14	− **.39**^**†**^	− .17	− .16	− .05	− .13
4	UPPS-P: lack premeditation	***.24***^***†***^	***.52***^***†***^	− *.03*	–	**.69**^**†**^	.18	**.36**^**†**^	**.37**^**†**^	.15	− **.53**^**†**^	− .13	− .15	.00	− .04
5	UPPS-P: lack perseverance	***.26***^***†***^	***.54***^***†***^	*.22*	***.69***^***†***^	–	.04	**.34**^**†**^	**.37**^**†**^	**.33**^**†**^	− **.64**^**†**^	− .15	− .16	.00	.02
6	UPPS-P: sensation seeking	*.15*	*.00*	*.23*	***.26***^***†***^	*.14*	–	**.45**^**†**^	**.33**^**†**^	− .15	− .23	− **.36**^**†**^	− .18	.10	− .02
7	UPPS-P: positive urgency	***.31***^***†***^	***.37***^***†***^	*.06*	***.28***^***†***^	*.18*	***.29***^***†***^	–	**.77**^**†**^	.17	− **.47**^**†**^	− .09	− .13	.01	.00
8	UPPS-P: negative urgency	***.25***^***†***^	***.69***^***†***^	*.07*	***.42***^***†***^	*.22*	*.16*	***.70***^***†***^	–	**.28**^**†**^	− **.53**^**†**^	− .03	− .09	.06	.07
9	TCI-R: harm avoidance	*.04*	***.59***^***†***^	*.21*	*.08*	***.27***^***†***^	− ***.26***^***†***^	*.22*	***.55***^***†***^	–	− **.44**^**†**^	.05	− .03	− .04	.16
10	TCI-R: self-directedness	− ***.37***^***†***^	− ***.80***^***†***^	− *.08*	− ***.62***^***†***^	− ***.57***^***†***^	− *.23*	− ***.59***^***†***^	− ***.71***^***†***^	− ***.44***^***†***^	–	.16	.15	.02	− .05
11	Chronological age (years-old)	− *.02*	− *.09*	*.19*	− ***.43***^***†***^	− *.19*	− *.10*	*.02*	− *.01*	− *.09*	*.18*	–	**.42**^**†**^	.10	− .08
12	Onset of gambling (years-old)	− ***.42***^***†***^	− ***.26***^***†***^	− *.08*	− ***.25***^***†***^	− *.04*	− *.18*	− ***.25***^***†***^	− ***.25***^***†***^	*.12*	*.07*	***.32***^***†***^	–	− .14	− .01
13	SLE: lifespan total	***.33***^***†***^	***.45***^***†***^	*.16*	*.14*	*.00*	− *.10*	*.22*	***.49***^***†***^	*.11*	− ***.33***^***†***^	***.30***^***†***^	− *.08*	–	− .06
14	Social position index	− *.04*	*.10*	− *.22*	*.13*	*.04*	− ***.45***^***†***^	*.02*	*.14*	*.18*	− *.12*	*.05*	*.20*	*.12*	–

Upper part of the table: estimations in the men subsample. Lower part of the table (Italic font): estimations in the women subsample.

DSM, Diagnostic and Statistical Manual of Mental Disorders; GRCS, gambling-related cognitions scale; PHQ, patient health questionnaire; SLE, stressful life events; TCI-R, temperament and character inventory-revised; UPPS-P, impulsive behavior scale.

^†^Bold: effect size into the mild-medium (|*R*|> 0.24) to high-large (|*R*|> 0.37) range.

The bivariate correlation estimation among females (lower part of Table 2, Italic font) showed that higher GD severity was related to higher scores in depression, cognitive bias, impulsivity, with lower scores in self-directedness, earlier onset of the gambling activity, and higher number of SLE lifespan. A worse depression state was reported for females with higher impulsivity levels, higher harm avoidance, lower self-directedness, younger onset of gambling, and higher number of SLE. There were no significant associations between cognitive bias and the other variables of the study.

### Pathways analyses

Separate path analyses (for men and women) were obtained through the next procedure: (a) firstly a complete path-diagram structure with all the variables considered in the correlational analysis (Table 2) was tested; (b) next, all non-significant coefficients were deleted to achieve a more parsimonious model; (c) fitting indexes for the finalistic model containing only significant coefficients (*p* < 0.05) or quasi-significant coefficients (*p* < 0.10) were obtained; and (d) the final model coincided with the finalistic model in the case of adequate goodness-of-fit, while a reviewed model was tested in the case of non adequate fitting. It should be noted that path analyses can be used for both exploratory and confirmatory modeling, and therefore it allows to theory testing and theory development^54^. The procedure used in this study is precisely justified in the exploratory nature of the SEM: starting from an initial model considering the largest number of variables that empirical evidences suggest that could contribute into the direct effects and the mediational links, and next re-adjust to obtain a most parsimonious model^55^. Retaining both significant and quasi-significant coefficients in the different steps is also recommended in multivariate models generated for exploring possible relationships rather than testing for a priori hypothesized paths, since fixing a p-value lower than 0.05 could result in a poor power or sensitivity [methodological studies show that an useful procedure is to screen complex relationships using a p-value of 0.10 (or even higher depending on the sample size), and next to investigate each final parameter to determine if it is substantively relevant]^56^.

**Figure 1:**
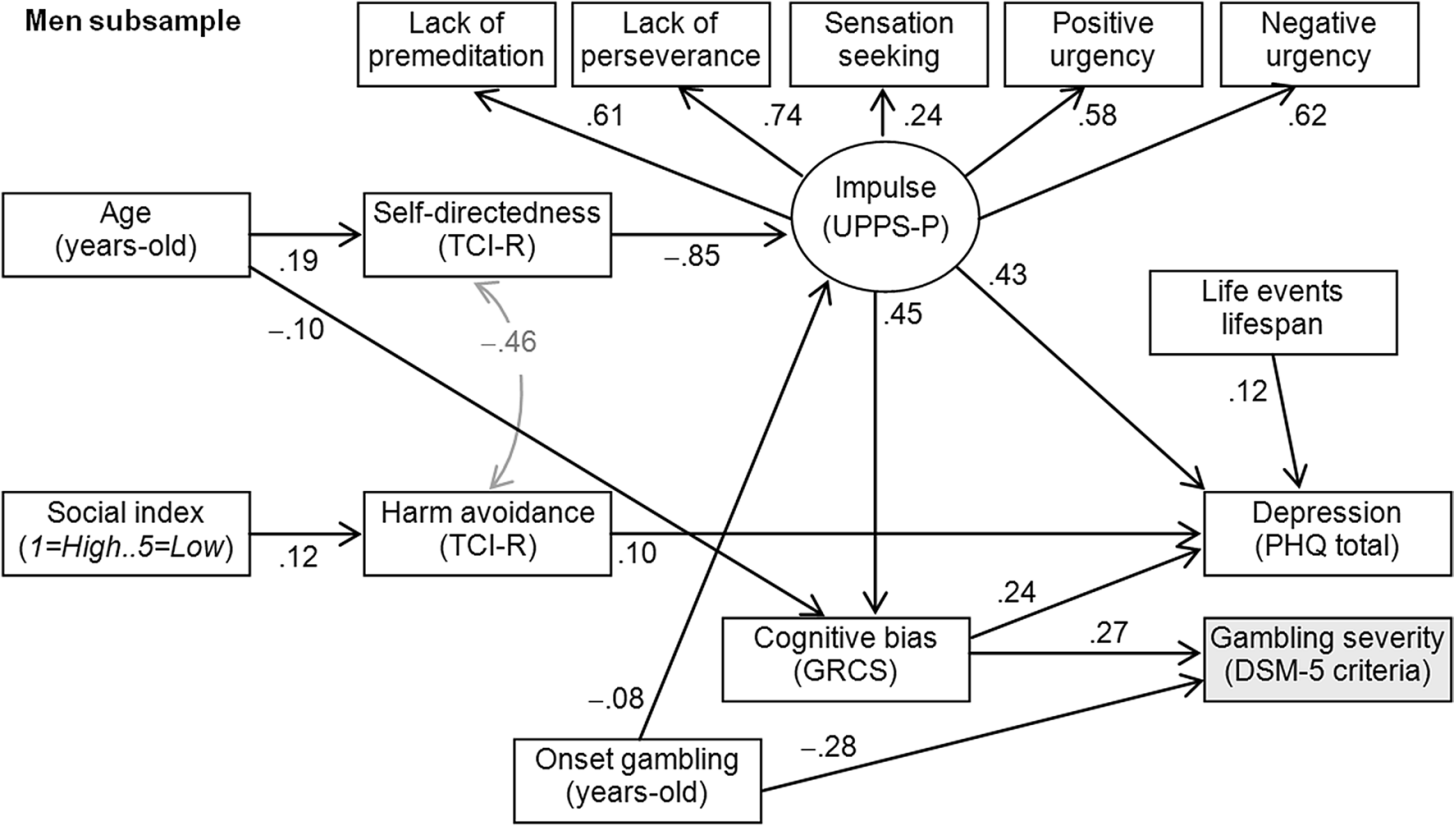
Path-diagram (men subsample): standardized coefficients. *Note*: Only significant coefficients are plotted. Grey color: covariance coefficients.

Figure 1 includes the final path-diagram with the standardized coefficients in the SEM obtained for the male subsample [only significant coefficients are plotted for easier interpretation; complete results for the SEM are reported in the Table S1 (supplementary material)]. This model corresponded to the finalistic model of the path analysis procedure, since goodness-of-fit was achieved (all the fitting indexes were in the adequate range: RMSEA = 0.044, CFI = 0.975, TLI = 0.961, SRMR = 0.046), and global predictive capacity was CD = 0.297 (around 30%). To limit the number of indicators, a latent variable measuring the impulsivity level was defined with the UPPS-P scale scores: all the measurement coefficients were positive and statistically significant, as such that higher levels in this latent variable are indicative of higher impulsivity levels. The results of the pathways showed that gambling severity among males was directly related to higher cognitive bias and younger age of onset of the gambling activity. The cognitive bias level was also a mediator variable between age and gambling level: younger chronological age was related to higher cognitive bias, which increased the likelihood of higher number of DSM-5 criteria for GD. Depression level did not correlate with the gambling severity in the male subsample, but a higher number of depressive symptoms was directly related with a higher number of SLE lifespan, higher impulsivity levels, higher harm avoidance score, and higher cognitive bias related to the gambling activity. Different mediational paths also explained the depression levels among males: (a) as the younger the age was the lower the self-directedness level: and this profile was related to the higher impulsivity levels which associated with worse depressive state; (b) higher impulsivity levels also mediated between younger onset of the gambling activity and the higher depression scores; (c) younger chronological age increased the cognitive bias level, which correlated with the higher depression symptoms; and (d) lower social position indexes were related to higher levels in harm avoidance, which correlated with the higher depression levels.

**Figure 2:**
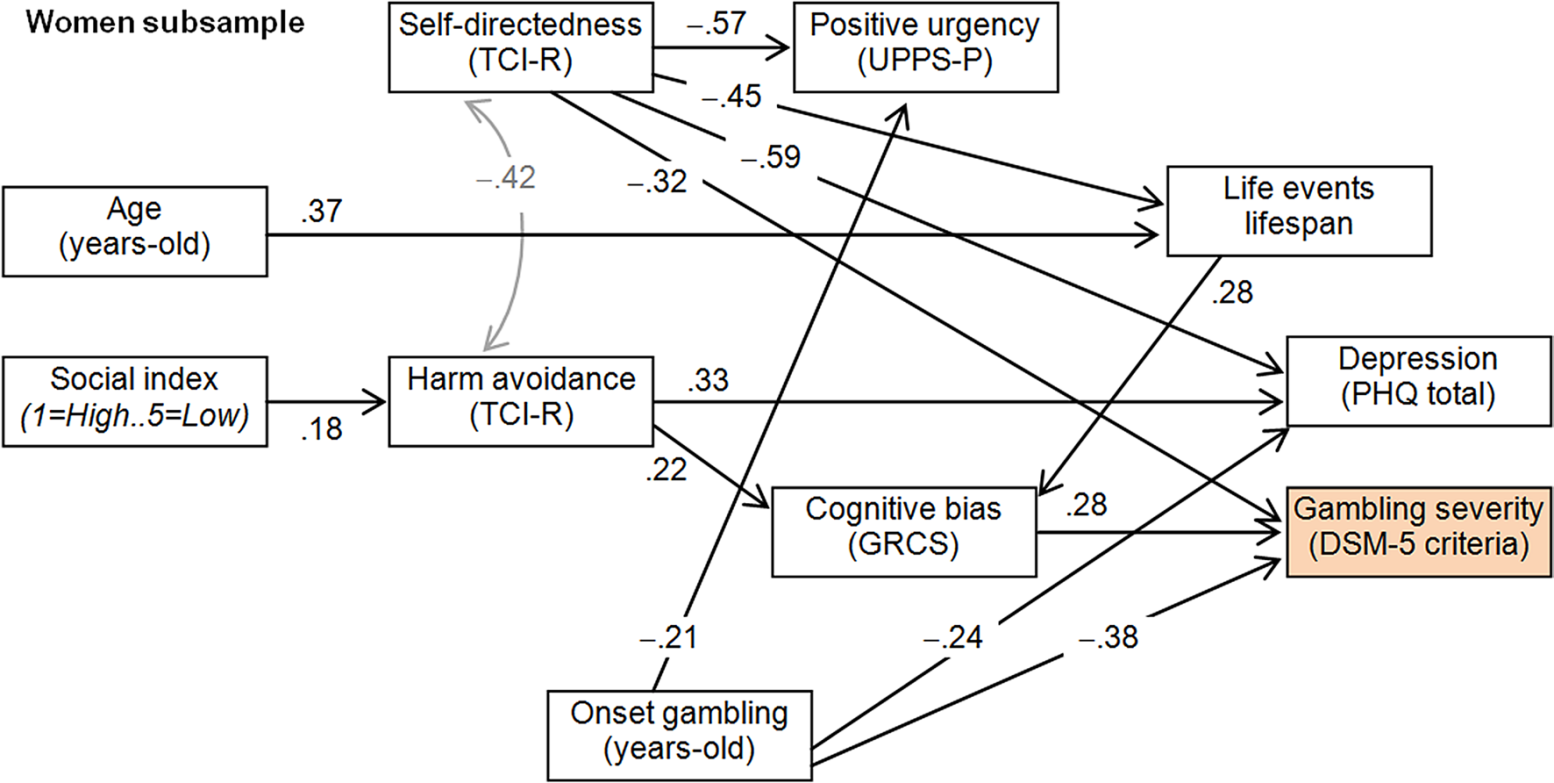
Path-diagram (women subsample): standardized coefficients. *Note:* Only significant coefficients are plotted. Grey color: covariance coefficients.

Figure 2 includes the path-diagram for the SEM obtained among females (only significant coefficients are plotted, and complete results are reported in the Table S1, supplementary material). This final model was a reviewed version of the finalistic version obtained in the procedure previously described for the path analysis, since no fitting was achieved considering the latent variable defined for the impulsivity construct. This final model achieved goodness-of-fit (RMSEA = 0.065, CFI = 0.962, TLI = 0.939, SRMR = 0.075), and global predictive capacity was CD = 0.491 (around 50%). For the female subsample it was not possible to include a latent variable, with the global impulsivity level being measured based on the UPPS-P scores due the lack of adequate fitting. In addition, only one measure of the impulsivity level was retained in the final model: the positive urgency score, since it had previously achieved the highest correlation with the GD severity (as shown in Table 2), and the other UPPS-P scales did not reach adequate fitting in the alternative candidate models tested in the study. Results of the SEM obtained for the females indicated that gambling severity was directly related to lower self-directedness level, higher cognitive bias related to gambling, and younger onset of the gambling activity. Two mediational links were also related to increasing the gambling severity: a) lower self-directedness scores related to a higher number of SLE lifespan, and this profile was correlated with the higher cognitive bias predicting more severe gambling activity; and b) lower social status was related to higher harm avoidance levels, and this profile also characterized individuals with higher cognitive bias, and therefore, was a predictor of higher gambling severity. As regards the depression levels, this mood factor was not associated with the number of DSM-5 criteria for GD, but it was related to lower levels in the self-directedness trait, higher levels in the harm avoidance domain, and younger age onset of the gambling behaviors.

## Discussion

To our knowledge, this is the first study examining the mediational role of a set of sociodemographic, personality and clinical variables on GD severity stratified by sex. The results of this study showed significant associations between different variables and gambling severity, but in a different way for males and females.

Initially, compared to females, males presented with a higher number of DSM-5 criteria for GD, with lower social position index scores, younger age, early onset of the gambling, higher impulsivity levels, lower level in the self-directedness personality trait, and a lower SLE lifespan.

Despite males showing higher levels of GD severity, severity in both groups was higher with higher levels of negative mood-related symptoms, cognitive bias, impulsivity, lower self-directedness, and with a younger age of gambling activity onset. Higher severity levels were also associated with a higher number of SLE in females. However, all these variables related differently in the final mediation paths, with some of them showing indirect and direct mediational effects on GD severity.

The resulting path model for males showed two variables directly related to gambling severity; higher cognitive bias and younger age of onset. In the case of females, a third variable was included, with a lower self-directedness level also directly related to gambling severity. A clear relationship between GD age of onset and GD severity has been previously reported^57–60^. Notably, the current paths by sex are partially in agreement with previous literature, reporting a strong relationship between gambling initiation, severity of the disorder, and age of onset for GD among males seeking treatment, compared to females^22,40,61^. However, in the current study, results showed that the relationship between GD onset age-GD severity is even stronger in females (magnitudes of − 0.38 vs − 0.28). The female group started to gamble significantly later than the male subgroup. This may be related to the fact that despite females normally starting gambling later in life compared to males, the progression from first stages to severe pathological phases is faster^62^. The relationship between age and SLE in females may probably be reflecting gambling as a form to counteract all the negative emotionality accumulated over time. In this sense, they begin to gamble after stressful situations (frustration, lack of satisfaction, etc.). Gambling would appear as an “antidepressant” that quickly becomes pathological in females. All in all, the discrepancies between our model and past findings regarding the contribution of sex into the relationship between the gambling severity with the onset age may be related to the different composition of the samples. Our study analyzed data provided by treatment-seeking patients, and the quick progression from occasional gambling to problematic/disordered gambling among females may be particularly exacerbated in treatment-seeking women. In this sense, our study contributes to highlight the potential relevance of the patients’ sex (and its intrinsic characteristics, some discussed here and possibly others unknown) into the GD profile. As we have observed, sex could modulate the effects of chronological age and onset age within the GD phenotypes, in the sense that males do not necessarily present higher likelihood for problematic gambling and/or more severe gambling behaviour compared to women, even when the gambling activities began at earlier ages. Our study also evidenced that within women GD age of onset appeared negatively related to both mood problems (depressive symptoms) and impulse positive urgency, suggesting that: the younger age onset the highest the depression and the positive urgency levels.

Cognitive bias, described as an irrational, simplified, or deviated thought from "normal" decisional outcomes^63^, and in this case involving a suboptimal ability to predict gambling consequences^64^, near-misses, and an “illusion of control”, among others^65^, has been previously found to achieve the best relevance for GD severity clustering in males^13^. Both males and females showed similar levels of cognitive distortion, but while this variable mediated the weight of age of participants and impulsive traits in males on gambling activity severity, cognitive bias mediated harm avoidance and SLE effects on GD severity in females. Curiously, harm avoidance mediated, in turn, social index effects on cognitive bias in females only, in the sense the lower the education and social position levels, the more the cognitive bias, and thus a probable propensity to illusion or unrealistic perceptions of control. In light of this puzzle, normally those who present lower educational and social status present worse adulthood outcomes, including, apart from an economic and emotional distress, a lower sense of control^66^. This may be partially explained because subjects with higher educational and social status are more often able to start a chain of successes than those of low social status (probably past success begets future success and past failure begets future failure), by using personal resources like income and education, thus increasing the sense of control and self-efficacy. In the current study, it seems that among those holding such illusions, the indirect mediation effects of these irrational thoughts about education and social position effects on GD severity are present (via harm-avoidance) only in women. The educational and social opportunities and casuistry of this part of the sample should require further exploration as, paradoxically, the prevalence of low social position cases where higher in men, thus suggesting that the influence of education and social status seems to be higher in women gamblers, in this case.

Cognitive bias, as well as impulsivity, are critical variables contributing to pathological gambling^23,67–69^. There is a clear link between cognitive dysfunctions and impulsivity in gambling, with impaired decision-making impulsive thoughts contributing to severity of GD^70,71^. The present results show that males tend to show more thoughtless behaviors related to cognitive interpretations than females. Current data suggest that maladaptive thoughts (cognitive bias) in females are mediating for the increase in the GD levels, probably avoiding the use of adaptive behavioral coping strategies to deal with stressors and manage daily problems. Cognition bias influencing GD severity in females seems to be related to how individuals proactively engage emotional regulation strategies to cope with daily life events, while males cognitive bias is directly related to engaging premature, inappropriate behaviors without planning the possible bad consequences^72^. Interestingly, impulsivity and adaptive coping strategies have been considered as independent^73^. In this vein, cognitive bias seems to be an independent variable compared to other personality and socio-demographic variables influencing GD severity. Cognitive bias implies a deviation from rationality directly affecting the act of decision-making in gambling, where desirable options should be decided, with an inability to predict consequences, so probably gamblers try to justify their biased beliefs about gambling outcomes and controllability. So, cognitive distortion appears as an abnormal emotion regulation directly linked to the act of gamble and its severity. People with GD exhibit distorted cognitions and superstitious beliefs more often than the general population to justify the act of gambling, using more often than controls maladaptive emotion regulation strategies to manage negative emotional states directly related to their acts^74^. In this sense, one could speculate that females try to engage, without success, “(mal)adaptive” strategies in a more proactive way than males, with cognitive bias being a mediator of these. Harm avoidance levels in the current subsamples agree with a previous study in a sample of gambler males, showing elevated harm avoidance levels compared to controls^75^; however the authors did not compare the male results with a female population. Current data have an added value, as they compare the moderator effect of sex on all these variables. Of note here, we cannot neglect the fact that cognitive biases mediate systematically, and only in males both emotional and motivational aspects of impulsivity (sensation seeking, positive and negative urgency) as well as its purely cognitive aspects (lack of perseverance and premeditation). Only the positive urgency in females appears in the path model, but without a direct or indirect influence over GD severity.

Anxiety-related processes (SLE) have a different role in the female and male paths. Stressful life events mediate the effects of age and self-directedness in females, and in turn associate with cognitive bias. In males, SLE are uniquely and directly related to mood processes. It has been reported that maladaptive coping can be the result of a reaction to stressful situations, and the current data show that stress management indirectly influences GD severity only in females through a bias in cognition. The literature is unclear as regards the relationship between gambling and SLE. Contrary to current data, some authors postulate that stress may influence gambling behavior only in some subgroups of individuals under high stress circumstances, especially males^76^, however males represented 33% of participants with gambling problems, while only 3% of females reported gambling problems in that sample. Others attribute perceived stress a moderator role between gambling severity and some forms of psychopathology, especially substance use and any Cluster-B personality disorder (which represents an erratic and emotional cluster)^77^. Differences in type of sample (adolescent versus adult populations, non-treatment-seeking versus treatment-seeking participants, levels of stress, among others) may also account for the differences compared to those of the Lightsey and Hulsey study^76^.

As previously mentioned, the SLE directly associated with depression in the male subsample. Depression levels did not appear in the path models as a factor related to gambling severity in any subsample. The exact nature of the relationship between gambling severity and depression is not clear in the literature. Previous works point out a co-occurrence between depression and pathological gambling symptoms^78,79^, with gamblers with comorbid depression having more severe problems, or with some mood-related sub-aspects, such as rumination, prolonging or intensifying the relationship between depression and problem gambling. A very recent mediational study found that deficits in non-acceptance, goals, strategies, and clarity in emerging adult gamblers, mediated the relationship between problem gambling and depression^80^. Coping was also found to predict pathological gambling and anxiety and depressive symptomatology^81^. Our current set of variables did not consider detailed coping strategies as a link between depressive symptomatology and gambling severity, but current correlational results between depression and GD severity need to be framed within a comorbid context, as we found that GD severity was higher for participants with higher depression level. With this in mind, researchers should focus on addressing comorbid depression symptomatology systematically in the assessment and evaluation of gambling problems, as pathological gambling seems to be frequently related to co-occurring mood-related factors^82–85^, which assume incorrect ways of regulating negative mood states^86–88^. How depression and impulsivity correlated to affect somehow GD severity deserves some attention. We found that GD severity in both groups was higher with higher levels of negative mood-related symptoms and impulsivity, with worse depression state for females with higher impulsivity levels. Having in mind that impulsivity implies “a predisposition toward rapid, unplanned reactions”^72^ or a failure to resist impulses^4^, it would seem that impulsivity should be a construct unrelated to depressive symptomatology, but, increasingly, impulsivity has been redefined as a multidimensional concept involving components not only related to actions, but also with unsuitable thoughts. I.e., cognitive impulsivity, beyond actions, involves an inability to evaluate the consequences of different events, with a consequent delay in gratification^89^, which may be involved in depressive processes. Impulsivity has been identified as a hallmark related to depression n different studies, with some depressed cases presenting elevated reflexive reactivity to emotions^90^, and with special relevance in the context of state dependent-related situations^91^.

In males, impulsivity level, harm avoidance, and cognitive bias directly mediated the effects of other factors (self-directedness level, onset of the gambling activity, chronological age, and social position indexes) in depression levels. In females, depression levels were directly related to lower levels in the self-directedness trait, and younger age onset of the gambling behaviors. In turn, in both male and female subsamples, harm avoidance acted as a mediator of social position in depressive-related symptoms. Maladaptive coping (harm avoidance) has been related to depressive symptomatology^92^ as an example of reactions to stress^93^.

While harm avoidance is reported as a maladaptive factor, high levels of self-directedness has been considered as a protective factor for developing depression and anxiety^94–96^. Coinciding with other authors, low scores in self-directness were included in the path models for males and females, affecting directly or indirectly GD severity. Two mediational ways were observed in females: (i) self-directness was directly and inversely related to GD, (ii) and it was related to higher number of SLE lifespan. This profile was also correlated with the higher cognitive bias predicting more severe gambling activity. In females, this factor also related to negative mood and positive urgency. In males, only an indirect way was observed, with impulsiveness mediating self-directness (the less the self-directness scores, the higher the impulsivity levels), and higher cognitive bias, which increased the likelihood of GD severity increases, mediating impulsivity. In both sexes, self-directness had a negative relationship with harm avoidance. In global terms, these results agree with previous literature on gamblers, reporting low levels of self-directness, together with high levels of harm avoidance^97–100^. Current paths show that self-directness, combined with high levels of harm avoidance, play an important role in gambling severity and associated depressive symptomatology, especially in females. Self-directness, a variable related to decision-making and planning behaviors, demonstrated a solid weight in the path model. Current results confirm the strong relationship between high gambling severity levels and low self-directness scores in participants seeking treatment^101^, especially in females^18^. In this respect, the contribution of the self-directness trait into the females’ path-diagram (compared to the contribution of this personality dimension into the males’ path-diagram) could be related to a higher involvement of a lack of stability to control everyday problems and stressful scenarios. The fact that self-directness directly affects severity only in females may account for the clear relationship between self-directness and neuroticism personality trait^102^. Neuroticism is highly correlated to harm avoidance trait in the Five-Factor Personality model of Cloninger^103^. At this point, low self-directness levels have been reported to be even more effective in determining problematic addictions than neuroticism per se. Bearing in mind sex differences in terms of neuroticism, with females showing systematically higher neuroticism levels than males^104^, our mediational path suggests that GD severity in females involves the influence of a maladjusted moody personality component related to anxiety and depression processes, showing a direct relationship between self-directness and both stress and depression components.

Overall, the SEM tested in the current study supported the idea of the existence of different mediational paths underlying mechanisms of gambling severity according to sex. The global predictive capacity of the resulting paths was higher for the female subgroup (50% vs 30%). Despite the lack of differences in the bivariate analysis comparing men and women for basic factors classically related with the gambling severity level [such as the global cognitive bias related to the gambling activity, depression, impulsivity or harm avoidance (as it was reported in Table 1)], the path analyses reveled that the role of these factors is different depending on the individuals’ sex. The results are clinically relevant, as they provide empirical evidence of distinct roles of sociodemographic, clinical and motivational factors, by sex, as well as different susceptibility to stress, mediating GD severity (SLE was implied in a mediational link only within women subsample).

### Limitations

The current study is not without limitations. Firstly, we cannot rule out the usual clinical effect involving the under-representation of females in gambling studies. The low frequency of women in the study may have different impacts in this work, such as the low statistical power to identify the presence of meaningful relationships between data and the generalization capacity. Low sample size for the female group have not allowed more rigorous methods for testing the potential differences between men and women in the path-diagram structures (such as a multi-group SEM to assess invariance by gender). Future research with higher sample sizes are required to legitimate the results obtained in this study, as well as to provide a more comprehensive picture of the GD profile, useful for developing adequate preventive and intervention plan centered in the individual idiosyncrasies.

Secondly, this sample represents treatment-seeking individuals, so there must be caution with the generality of results in population-based samples, random samples or studies carried out in other countries.

Thirdly, although we used a set of sociodemographic, clinical and personality variables possibly involved in GD severity, they probably are not enough to entirely describe mediational paths involved in gambling behavior, as some other clinical variables or sub-aspects not evaluated here may be mediating some of the relationships found here.

Fourth, since the data analyzed in this study was recruited through a cross-sectional design, and the nature of the research is clearly exploratory, causal interpretation cannot be directly assumed. Traditionally the cross-sectional study has been considered an undesired design that do not allow for causal diagrams. But current research suggest that this design can also be used to assess a theoretical causal structure since ultimately the uncertainty about the order of causation (ambiguous temporality) depends on the nature of the postulated cause and the measurement method^105,106^. Accordingly, it has been outlined that path analysis can be used for both exploratory and confirmatory modeling, and therefore it allows to theory testing and theory development^54,55^. In any case, future longitudinal research should assess the concrete role of the variables in the underlying mechanism of the GD profile.

### Strengths and implications

This study uses pathways analysis to explore the underlying mechanisms explaining the severity of the gambling behavior stratified by the patient sex, resulting, as mentioned above, in a group with a low sample size representation for females. While pathways analysis has been widely used in different areas of the behavioral science research, for a long time it has been considered that it required confirmatory modeling and large sample sizes as conditions. But recent studies show that these theoretical requirements seem to rely on outdated rules-of-thumb, and experts point out that this procedure can be used for both exploratory and confirmatory modeling, and therefore it allows theory testing and theory development^54^. Simulation analyses using Monte-Carlo procedures have been used to analyze the sample size requirements for some common types of SEMs, including variation by the number of factors, number of indicators, strength of the indicator loadings and the regressive paths and the amount of missing data per indicator^107^. The analysis performed with respect to the statistical power, a bias in the parameter estimates, and overall solution propriety, showed that the sample requirements were into a very broad range (from 30 to 460), depending on the analysis characteristics. But the most interesting thing is that, overall, solutions that met fitting at a given sample size, were stable relative to the results of the analysis at the next largest sample sizes. What seems to be more relevant here is the adequate goodness-of-fit, meaning that the use of pathways-analysis should be considered as strength of this work. It is true that the low female sample size affects the modeling, since the SEM obtained for this subsample has a low statistical power to identify potential relationships between the variables. Future studies involving a greater number of females are needed to confirm (or reject) the results obtained in this work.

Our results have also implications for the development of future research studies in the clinical area, focused in obtaining a more precise picture of the individual-characteristics implied in the treatment outcomes. Sex seems to be a relevant variable, and other personal and contextual features should be assessed to improve response to treatments, and diminish the risk of relapses and/or withdrawals.

## Supplementary information

Supplementary Table S1.

## Data Availability

Data cannot be shared publicly because of being part of a public hospital clinical database. Data are available from the Hospital Universitari de Bellvitge Institutional Data Access/Ethics Committee (IDIBELL; otri@idibell.cat) for researchers who meet the criteria for access to confidential data.
