# Supplementary material for: Contribution of sex on the underlying mechanism of the gambling disorder severity
Source: Sci Rep. 2020 Oct 30;10:18722. doi: 10.1038/s41598-020-73806-6 (PMC7599246; doi:10.1038/s41598-020-73806-6)
Supplement: Supplementary file 1 — Supplementary Table S1. [file 41598_2020_73806_MOESM1_ESM.docx]

Title. Contribution of sex on the underlying mechanism of the gambling disorder severity

**Authors and affiliations**

Susana Jiménez-Murcia^1,2,3,4^, Roser Granero*^1,5^, Mónica Giménez^2,6^, Amparo del Pino-Gutiérrez^2,7^, Gemma Mestre-Bach^1,2^, Teresa Mena-Moreno^1,2^, Laura Moragas^2^, Marta Baño^2^, Jéssica Sánchez-González^2^, Marta de Gracia^2^, Isabel Baenas-Soto^2^, S.Fabrizio Contaldo^2^, Eduardo Valenciano-Mendoza^2,6^, Bernat Mora-Maltas^2^, Hibai López-González^2^, José M. Menchón^2,3,6^, Fernando Fernández-Aranda^1,2,3,4^

*^1^Ciber Fisiopatología Obesidad y Nutrición (CIBERobn), Instituto Salud Carlos III, Madrid, Spain*

*^2^Department of Psychiatry, Hospital Universitari de Bellvitge, L'Hospitalet de Llobregat, Spain.*

*^3^Department of Clinical Sciences, School of Medicine, Universitat de Barcelona - UB, L'Hospitalet de Llobregat, Spain.*

*^4^Psychiatry and Mental Health Group, Neuroscience Program, Institut d'Investigació Biomèdica de Bellvitge - IDIBELL, L'Hospitalet de Llobregat, Spain.*

*^5^Department of Psychobiology and Methodology, Autonomous University of Barcelona, Barcelona, Spain*

*^6^Ciber Salud Mental (CIBERsam), Instituto de Salud Carlos III. Madrid, Spain*

*^7^Department of Public Health, Mental Health and Perinatal Nursing, School of Nursing, University of Barcelona, Barcelona, Spain.*

***Corresponding author:**

Roser Granero

Department of Psychiatry. Bellvitge University Hospital.

C/ Feixa Llarga s/n. C.P. 08907. Hospitalet de Llobregat. Barcelona. Spain.

Tel.: +34 93 260 79 88; Fax. +34 93 260 76 58.

*E-mail address*: Roser.Granero@uab.cat

*Table S1(supplementary material) Results obtained in the SEM: direct, indirect and total effects*

| **Men subsample** |
| --- |
|  |
| Direct effects \| Coef. Std. Err. z P>\|z\| Std. Coef. |
| TCI_SD \| |
| AGE \| .3326574 .0704365 4.72 0.000 .1895065 |
| DSM5_GDtotal \| |
| GRCStotal \| .0161864 .0024755 6.54 0.000 .2743175 |
| OnsetGAMES \| -.0644489 .0095148 -6.77 0.000 -.2832904 |
| PHQtotal \| |
| GRCStotal \| .0484886 .0089276 5.43 0.000 .2353724 |
| TCI_HA \| .049379 .0219308 2.25 0.024 .0912141 |
| UPPSP \| .19702 .0295037 6.68 0.000 .4328652 |
| SLElifespanTOT \| .0954127 .0299687 3.18 0.001 .1166851 |
| TCI_HA \| |
| Social \| 1.518788 .482338 3.15 0.002 .1232379 |
| UPPSP \| |
| TCI_SD \| -.5310616 .0556581 -9.54 0.000 -.8458246 |
| OnsetGAMES \| -.1254844 .0652697 -1.92 0.055 -.0719076 |
| GRCStotal \| |
| UPPSP \| 1 (constrained) .4526122 |
| AGE \| -.2434288 .102242 -2.38 0.017 -.0999679 |
| Indirect effects \| Coef. Std. Err. z P>\|z\| Std. Coef. |
| DSM5_GDtotal \| |
| TCI_SD \| -.008596 .0016081 -5.35 0.000 -.1050171 |
| UPPSP \| .0161864 .0024755 6.54 0.000 .1241595 |
| AGE \| -.0067997 .0020283 -3.35 0.001 -.0473244 |
| OnsetGAMES \| -.0020311 .0010998 -1.85 0.065 -.008928 |
| PHQtotal \| |
| TCI_SD \| -.1303802 .0119167 -10.94 0.000 -.4562358 |
| UPPSP \| .0484886 .0089276 5.43 0.000 .1065324 |
| AGE \| -.0551754 .0112314 -4.91 0.000 -.1099893 |
| Social \| .0749962 .0410215 1.83 0.068 .011241 |
| OnsetGAMES \| -.0308075 .0157377 -1.96 0.050 -.0387868 |
| UPPSP \| |
| AGE \| -.1766616 .041751 -4.23 0.000 -.1602893 |
| GRCStotal \| |
| TCI_SD \| -.5310616 .0556581 -9.54 0.000 -.3828306 |
| AGE \| -.1766616 .041751 -4.23 0.000 -.0725489 |
| OnsetGAMES \| -.1254844 .0652697 -1.92 0.055 -.0325463 |
|  |
|  |
| Total effects \| Coef. Std. Err. z P>\|z\| Std. Coef. |
| TCI_SD \| |
| AGE \| .3326574 .0704365 4.72 0.000 .1895065 |
| DSM5_GDtotal \| |
| GRCStotal \| .0161864 .0024755 6.54 0.000 .2743175 |
| TCI_SD \| -.008596 .0016081 -5.35 0.000 -.1050171 |
| UPPSP \| .0161864 .0024755 6.54 0.000 .1241595 |
| AGE \| -.0067997 .0020283 -3.35 0.001 -.0473244 |
| OnsetGAMES \| -.0664801 .0096094 -6.92 0.000 -.2922184 |
| PHQtotal \| |
| GRCStotal \| .0484886 .0089276 5.43 0.000 .2353724 |
| TCI_SD \| -.1303802 .0119167 -10.94 0.000 -.4562358 |
| TCI_HA \| .049379 .0219308 2.25 0.024 .0912141 |
| UPPSP \| .2455086 .027832 8.82 0.000 .5393976 |
| AGE \| -.0551754 .0112314 -4.91 0.000 -.1099893 |
| Social \| .0749962 .0410215 1.83 0.068 .011241 |
| OnsetGAMES \| -.0308075 .0157377 -1.96 0.050 -.0387868 |
| SLElifespanTOT \| .0954127 .0299687 3.18 0.001 .1166851 |
| TCI_HA \| |
| Social \| 1.518788 .482338 3.15 0.002 .1232379 |
| UPPSP \| |
| TCI_SD \| -.5310616 .0556581 -9.54 0.000 -.8458246 |
| AGE \| -.1766616 .041751 -4.23 0.000 -.1602893 |
| OnsetGAMES \| -.1254844 .0652697 -1.92 0.055 -.0719076 |
| GRCStotal \| |
| TCI_SD \| -.5310616 .0556581 -9.54 0.000 -.3828306 |
| UPPSP \| 1 (constrained) .4526122 |
| AGE \| -.4200904 .1074708 -3.91 0.000 -.1725168 |
| OnsetGAMES \| -.1254844 .0652697 -1.92 0.055 -.0325463 |

| **Women subsample** |
| --- |
| Direct effects \| Coef. Std. Err. z P>\|z\| Std. Coef. |
| SLElifespanTOT \| |
| TCI_SD \| -.2168469 .0659433 -3.29 0.001 -.4485193 |
| AGE \| .3855809 .1443203 2.67 0.008 .366629 |
| TCI_SD \| |
| AGE \| .3193205 .3114886 1.03 0.305 .1467946 |
| Social \| -3.312756 4.000968 -0.83 0.408 -.1302839 |
| GRCStotal \| |
| SLElifespanTOT \| .6998675 .3851246 1.82 0.069 .2808605 |
| DSM5_GDtotal \| |
| TCI_SD \| -.0337901 .0136855 -2.47 0.014 -.3193612 |
| GRCStotal \| .0248179 .0114391 2.17 0.030 .2825886 |
| OnsetGAMES \| -.0925889 .0314589 -2.94 0.003 -.3827414 |
| TCI_HA \| |
| Social \| 2.449871 2.11812 1.16 0.247 .1821112 |
| Positive_UR \| |
| TCI_SD \| -.2262718 .0496377 -4.56 0.000 -.5732252 |
| OnsetGAMES \| -.1917115 .1141819 -1.68 0.093 -.2124209 |
| PHQtotal \| |
| SLElifespanTOT \| .0794734 .0474353 1.68 0.094 .1381653 |
| TCI_SD \| -.1638065 .0246136 -6.66 0.000 -.5890284 |
| TCI_HA \| .1746424 .0448584 3.89 0.000 .3322482 |
| OnsetGAMES \| -.1548208 .0487718 -3.17 0.002 -.2434943 |
| Indirect effects \| Coef. Std. Err. z P>\|z\| Std. Coef. |
| SLElifespanTOT \| |
| AGE \| -.0692437 .0707515 -0.98 0.328 -.0658402 |
| Social \| .7183609 .8946773 0.80 0.422 .0584349 |
| GRCStotal \| |
| TCI_SD \| -.1517641 .095417 -1.59 0.112 -.1259714 |
| AGE \| .2213941 .1641913 1.35 0.178 .0844797 |
| Social \| .5027575 .6845515 0.73 0.463 .016412 |
| DSM5_GDtotal \| |
| SLElifespanTOT \| .0173692 .0124679 1.39 0.164 .079368 |
| TCI_SD \| -.0037665 .0029362 -1.28 0.200 -.0355981 |
| AGE \| -.0052953 .0133925 -0.40 0.693 -.0230075 |
| Social \| .1244158 .157122 0.79 0.428 .0462455 |
| Positive_UR \| |
| AGE \| -.0722532 .0722414 -1.00 0.317 -.0841464 |
| Social \| .7495834 .9201192 0.81 0.415 .074682 |
| PHQtotal \| |
| TCI_SD \| -.0172336 .0115443 -1.49 0.135 -.0619698 |
| AGE \| -.0271664 .0605655 -0.45 0.654 -.0449077 |
| Social \| 1.027593 .9483866 1.08 0.279 .1453207 |
| Total effects \| Coef. Std. Err. z P>\|z\| Std. Coef. |
| SLElifespanTOT \| |
| TCI_SD \| -.2168469 .0659433 -3.29 0.001 -.4485193 |
| AGE \| .3163372 .1572791 2.01 0.044 .3007888 |
| Social \| .7183609 .8946773 0.80 0.422 .0584349 |
| TCI_SD \| |
| AGE \| .3193205 .3114886 1.03 0.305 .1467946 |
| Social \| -3.312756 4.000968 -0.83 0.408 -.1302839 |
| GRCStotal \| |
| SLElifespanTOT \| .6998675 .3851246 1.82 0.069 .2808605 |
| TCI_SD \| -.1517641 .095417 -1.59 0.112 -.1259714 |
| AGE \| .2213941 .1641913 1.35 0.178 .0844797 |
| Social \| .5027575 .6845515 0.73 0.463 .016412 |
| DSM5_GDtotal \| |
| SLElifespanTOT \| .0173692 .0124679 1.39 0.164 .079368 |
| TCI_SD \| -.0375566 .0138609 -2.71 0.007 -.3549593 |
| GRCStotal \| .0248179 .0114391 2.17 0.030 .2825886 |
| AGE \| -.0052953 .0133925 -0.40 0.693 -.0230075 |
| Social \| .1244158 .157122 0.79 0.428 .0462455 |
| OnsetGAMES \| -.0925889 .0314589 -2.94 0.003 -.3827414 |
| TCI_HA \| |
| Social \| 2.449871 2.11812 1.16 0.247 .1821112 |
| Positive_UR \| |
| TCI_SD \| -.2262718 .0496377 -4.56 0.000 -.5732252 |
| AGE \| -.0722532 .0722414 -1.00 0.317 -.0841464 |
| Social \| .7495834 .9201192 0.81 0.415 .074682 |
| OnsetGAMES \| -.1917115 .1141819 -1.68 0.093 -.2124209 |
| PHQtotal \| |
| SLElifespanTOT \| .0794734 .0474353 1.68 0.094 .1381653 |
| TCI_SD \| -.1810401 .0240985 -7.51 0.000 -.6509983 |
| TCI_HA \| .1746424 .0448584 3.89 0.000 .3322482 |
| AGE \| -.0271664 .0605655 -0.45 0.654 -.0449077 |
| Social \| 1.027593 .9483866 1.08 0.279 .1453207 |
| OnsetGAMES \| -.1548208 .0487718 -3.17 0.002 -.2434943 |
